# Relationship between ABO blood groups and cardiovascular disease in type 1 diabetes according to diabetic nephropathy status

**DOI:** 10.1186/s12933-020-01038-z

**Published:** 2020-05-19

**Authors:** Erika B. Parente, Valma Harjutsalo, Markku Lehto, Carol Forsblom, Niina Sandholm, Per-Henrik Groop, on behalf of the FinnDiane Study Group

**Affiliations:** 1 Folkhälsan Institute of Genetics, Folkhälsan Research Center, Helsinki, Finland; 2 Faculdade de Ciências Médicas da Santa Casa de São Paulo, São Paulo, Brazil; 3 Research Program for Clinical and Molecular Metabolism, Faculty of Medicine, University of Helsinki, Helsinki, Finland; 4 Abdominal Center, Nephrology, University of Helsinki and Helsinki University Hospital, Helsinki, Finland; 5 National Institute for Health and Welfare, Chronic Disease Prevention Unit, Helsinki, Finland; 6 Department of Diabetes, Central Clinical School, Monash University, Melbourne, Australia

**Keywords:** Type 1 diabetes, Diabetic nephropathy, Cardiovascular disease, Blood group

## Abstract

**Background:**

ABO blood groups have previously been associated with cardiovascular disease (CVD) in the general population. This study aimed to investigate the potential relationship between ABO blood groups and CVD in individuals with type 1 diabetes according to diabetic nephropathy (DN) status.

**Methods:**

Adults with type 1 diabetes (4531 individuals) from the FinnDiane Study were evaluated. DN was determined by two out of three measurements of urinary albumin excretion rate. Albuminuria was defined as an excretion rate above 20 µg/min. CVD events were identified by linking the data with the Finnish Care Register for Health Care and the Finnish Cause of Death Register. Follow-up ranged from the baseline visit until a CVD event, death or the end of 2017. The impact of ABO blood groups on CVD risk was estimated by multivariable Cox-regression analyses adjusted for traditional risk factors.

**Results:**

At baseline, the median age was 38.5 (IQR 29.2–47.9) years, 47.5% were female and median duration of diabetes was 20.9 (11.4–30.7) years. There were 893 incident ischemic heart disease (IHD) events, 301 ischemic strokes (IS), and 415 peripheral artery disease (PAD) events during a median follow up of 16.5 (IQR 12.8–18.6) years. The A blood group showed the highest risk of IHD versus the O blood group, when microalbuminuria was present. Comparing the population with microalbuminuria with those with normoalbuminuria, only the A blood group elevated the risk of IHD. This increased risk was neither explained by the *FUT2* secretor phenotype nor by the A-genotype distribution. The risk of IS or PAD was no different among the ABO blood groups regardless of diabetic nephropathy stage.

**Conclusion:**

The A blood group is a risk factor for IHD in individuals with type 1 diabetes and microalbuminuria.

## Background

Cardiovascular disease (CVD) is the major cause of death among people with diabetes worldwide and the ABO blood groups have been associated with CVD in several studies [1–3]. The first time the ABO blood groups were shown to be associated with CVD was in 1962 [4] when the A and B blood groups were linked to ischemic heart disease (IHD). Recently, it was described that ABO blood groups are associated with increased cardiovascular risk in individuals with familial hypercholesterolemia [5]. The majority of previous studies have indicated that the O blood group confers the lowest risk of thrombotic events, although the group with the highest risk is still controversial and depending on the studied population [1–3, 6].

The reasons why the ABO blood group is a risk factor of CVD is still under investigation. ABO antigens are not expressed only on the surface of red blood cells but also on epithelial and endothelial cells, T-cells, B-cells and platelets [8]. These antigens might also be found in the circulation and body secretions, if the individual has the *FUT2* gene secretor phenotype [8]. The interaction between ABO blood antigens and adhesion molecules, such as the soluble InterCellular Adhesion Molecule 1 (sICAM-1), may differ depending on the A blood group subtypes A1 or A2, which interferes with the leucocyte endothelium adhesion [9]. Different levels of the von Willebrand factor (vWF) [10] and HDL-cholesterol concentrations [11] have also been suggested to explain the relationship between ABO blood groups and CVD risk.

CVD is a major cause of premature mortality in individuals with type 1 diabetes, especially if diabetic nephropathy (DN) is present [12–14]. Acute diabetic complications drive the mortality rate during the first years of living with type 1 diabetes, but IHD becomes the main cause of premature mortality of those with longer duration of diabetes [15]. Although there are several well-known CVD risk factors in type 1 diabetes, the potential impact of the ABO blood groups on this risk has never been studied in this population. This study therefore aimed to explore this unanswered question taking different DN stages into account.

## Methods

### Research design

This is an observational prospective study to evaluate the relationship between ABO blood groups and CVD in type 1 diabetes according to DN stage as part of the ongoing Finnish Diabetic Nephropathy (FinnDiane) Study, which is a nationwide, prospective, multicenter study aiming to identify risk factors for type 1 diabetes complications. The CVD risk of ABO blood groups was compared according to the various stages of DN.

### Study population

The FinnDiane Study has since 1997 recruited and characterized individuals with type 1 diabetes 18 years or older from 93 centers across Finland and the recruitment of new study participants is still ongoing. This analysis included 4531 individuals with type 1 diabetes with urinary albumin excretion rate (UAER) data and information on ABO blood groups available. Type 1 diabetes was defined as age at onset of diabetes under 40 years and permanent insulin treatment initiated within a year from the diabetes diagnosis. The study protocol followed the principles of the Declaration of Helsinki as revised in 2000 and was approved by the Ethical Committee of Helsinki and Uusimaa Hospital District. Written informed consents were obtained from each FinnDiane participant. The baseline visit occurred between the years 1997 and 2015 and at which the participants underwent a thorough clinical examination, blood and urine samples were collected and several questionnaires including specific questions about lifestyle habits were completed by the participants.

### DN stage

The stage of DN was based on the individuals’ urinary albumin excretion rate (UAER) from timed overnight and 24 h urine (mg/24 h) collections. Normoalbuminuria was defined as a UAER < 20 µg/min or < 30 mg/24 h in at least two out of three urine samples. Microalbuminuria was defined as UAER ≥ 20 and < 200 µg/min or ≥ 30 and < 300 mg/24 h, macroalbuminuria as UAER ≥ 200 µg/min or ≥ 300 mg/24 h and end-stage renal disease (ESRD) as dialysis or kidney transplantation.

### ABO blood groups

The ABO blood groups were determined based on genetic variants coding for or tagging the O (rs8176719 deletion allele), B (rs8176746 A allele), and A (rs8176747 G allele) groups [16]. The A1 versus A2 subgroup was defined based on the rs8176750 C allele [17]. Secretor status was defined based on the *FUT2* rs601338 AG/GG genotypes [18]. Genotyping was done with Human Core Exome Bead Chips 12–1.0, 12–1.1 or 24–1.0 (Illumina, San Diego, CA, USA), with genotype imputation using the 1000 genomes reference panel, as described earlier [19]. The rs8176746 was directly genotyped, and the other variants were imputed with high quality (r^2^ ≥ 1.0); most likely genotypes were used for the imputed variants.

### CVD

With CVD events we refer to IHD, ischemic stroke (IS) and peripheral arterial disease (PAD). The corresponding international classification of disease (ICD) codes are listed in the Additional file 1: table S1. CVD events were identified by linking the data with the Finnish Care Register for Health Care and the Finnish Cause of Death Register. Participants that had had a CVD event before the baseline visit were excluded. The follow-up period ranged from the baseline visit until the occurrence of the first CVD event, death or the end of 2017.

**Table 1:** Baseline characteristics according to ABO blood groups

	O	A	B	AB	p
	n = 1333 (29.4%)	n = 2012 (44.4%)	n = 796 (17.6%)	n = 390 (8.6%)	value
Sex (female %)	46.5	47.5	48.0	50.3	0.62
Age (years)	38.0 (29.2–47.5)	38.2 (29.2–47.8)	39.2 (29.1–48.4)	39.3 (29.0–48.5)	0.81
Duration of diabetes (years)	21.5 (11.3–30.8)	20.4 (11.4–30.3)	21.3 (12.0–30.7)	21.0 (11.2–32.7)	0.47
HbA_1c_ (%)	8.4 ± 1.5	8.4 ± 1.5	8.4 ± 1.5	8.3 ± 1.4	0.46
Smoking history (%)	46.4	47.5	47.4	46.9	0.95
Body Mass Index (kg/m^2^)	25.1 ± 3.5	25.1 ± 3.8	25.0 ± 3.5	25.0 ± 3.9	0.81
Waist-to-hip ratio	0.87 ± 0.08	0.87 ± 0.09	0.87 ± 0.09	0.87 ± 0.08	0.71
Systolic blood pressure (mmHg)	134 ± 18	135 ± 19	134 ± 18	135. ± 20	0.44
Diastolic blood pressure (mmHg)	80 ± 10	80 ± 10	80 ± 10	79 ± 10	0.74
Total cholesterol (mmol/l)	4.90 ± 0.99	4.95 ± 1.01	4.87 ± 0.93	4.95 ± 0.95	0.17
HDL cholesterol (mmol/l)	1.32 ± 0.38	1.33 ± 0.39	1.35 ± 0.40	1.36 ± 0.41	0.25
Triglycerides (mmol/l)	1.06 (0.78–1.51)	1.03 (0.78–1.52)	1.01 (0.77–1.45)	1.04 (0.75–1.40)	0.16
Hs-CRP (mg/l)	4.5 ± 9.4	4.6 ± 9.4	4.2 ± 8.5	4.7 ± 9.0	0.64
DN groups					0.42
Normoalbuminuria (n, %)	862 (64.7)	1311 (65.2)	505 (63.4)	253 (64.9)	
Microalbuminuria (n, %)	178 (13.4)	251 (12.5)	109 (13.7)	44 (11.3)	
Macroalbuminuria (n, %)	199 (14.9)	272 (13.5)	124 (15.6)	55 (14.1)	
ESRD (n, %)	94 (7.0)	178 (8.8)	58 (7.3)	38 (9.7)	
Laser treatment (%)	34.7	31.9	35.2	34.3	0.22
Lipid lowering therapy (%)	13.0	14.6	11.6	15.0	0.36
Blood Pressure lowering therapy (%)	38.8	39.3	39.0	40.0	0.98
ACE/ARB users (%)	30.3	31.0	30.7	30.5	0.98
FUT2 Secretor (%)	81.4	82.7	82.4	83.5	0.72
Antibiotic purchases per person year	0.62 (0.30–1.46)	0.63 (0.28–1.33)	0.59 (0.26–1.34)	0.60 (0.28–1.30)	0.90
IHD (n, %)	82 (6.2)	132 (6.6)	49 (6.2)	30 (7.7)	0.50
Socioeconomic status					
Blue collar worker (%)	56.9	58.0	60.7	57.0	0.48
White collar worker (%)	32.6	30.2	30.4	32.3	
Others (%)	3.6	4.3	2.8	3.2	
Not known (%)	6.9	7.5	6.1	7.6	
Alcohol consumption (g/week)	48.0 (24.0–84.0)	48.0 (24.0–84.0)	42.0 (24.0–84.0)	36.0 (24.0–84.0)	0.06
Abstainers (%)	26.5	27.1	26.8	26.7	0.18
Missing data (%)	9.3	9.8	9.8	6.2	0.76

Data are given as mean ± SD or median and interquartile range

*HbA*_*1c*_ glycated hemoglobin, *Hs-CRP* high-sensitivity C-reactive protein, *DN* diabetic nephropathy, *ACE* angiotensin-converting enzyme inhibitor, *ARB* angiotensin receptor blockers, *HDL* high-density lipoprotein, *FUT2* fucosyltransferase-2, *IHD* ischemic heart disease

*p* value refers to ANOVA, Kruskal–Wallis test or χ2-test adjusted for age at baseline when applicable. Comparisons are made among ABO blood groups

### Utilization of antibiotic drugs

We used the Finnish Drug Prescription Register of antibiotic purchase per person per year to investigate the distribution of the number of infections per person per year among the ABO blood groups.

### Statistical analysis

Data on categorical variables are presented as frequencies, while continuous variables are shown as means (± standard deviation, SD) for normally distributed values and variables with skewed distribution as medians (interquartile ranges, IQR). Between-group comparisons were performed with Chi squared test, ANOVA for normally distributed continuous variables, otherwise by the Kruskal–Wallis test.

The impact of the ABO blood groups on CVD risk was estimated by multivariable Cox-regression analyses adjusted for the following traditional risk factors: age at diabetes diagnosis, body mass index (BMI), duration of diabetes, systolic blood pressure (SBP), HDL cholesterol, triglycerides, HbA_1c_, sex, severe diabetic retinopathy (defined as laser treatment) and history of smoking. Interaction between ABO blood groups and DN status was tested and since it was significant, the analyses were conducted separately for each DN group. However, the interaction between the ABO blood groups and sex was not significant and therefore these analyses were conducted by pooling men and women. The assumption of the proportional hazards was tested by Schoenfeld residuals against time and testing a non-zero slope by including time-covariate interactions. In case the assumption did not hold the interaction between the covariate and time was added to the final model. All analyses were performed with Statistical Analysis System version 9.4 (SAS Institute, Cary, NC, USA).

## Results

At baseline, the median age was 38.5 (IQR 29.2–47.9) years, 47.5% were female and the median duration of diabetes was 20.9 (11.4–30.7) years. There were 893 incident IHD events, 301 IS and 415 PAD events during 62,326 person-years for IHD, 68,137 for IS and 67,118 for PAD during a median follow up of 16.5 (IQR 12.8–18.6) years.

**Fig. 1:**
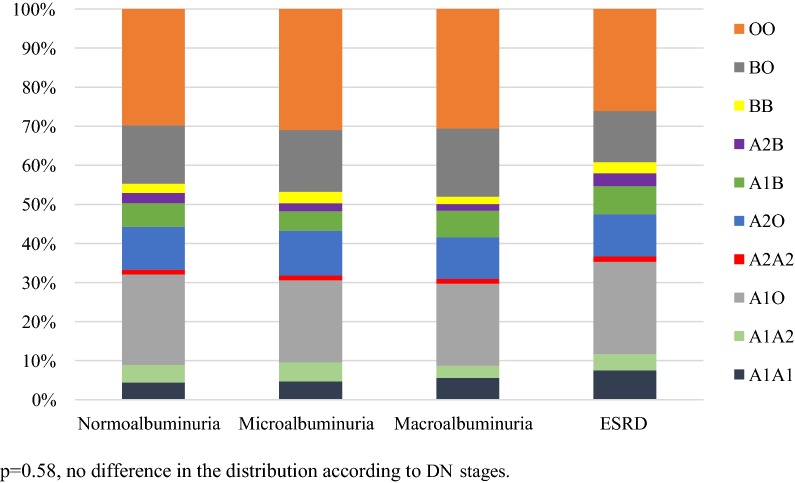
Distribution of ABO genotype by DN stages

Baseline characteristics such as the prevalence of IHD, FUT2 secretor phenotype, number of antibiotic purchases per person per year, serum hs-CRP level, DN stages and other well-known CVD risk factors were no different between the ABO blood groups (Table 1). ABO genotypes were also equally distributed according to the DN stages (Fig. 1).

**Fig. 2:**
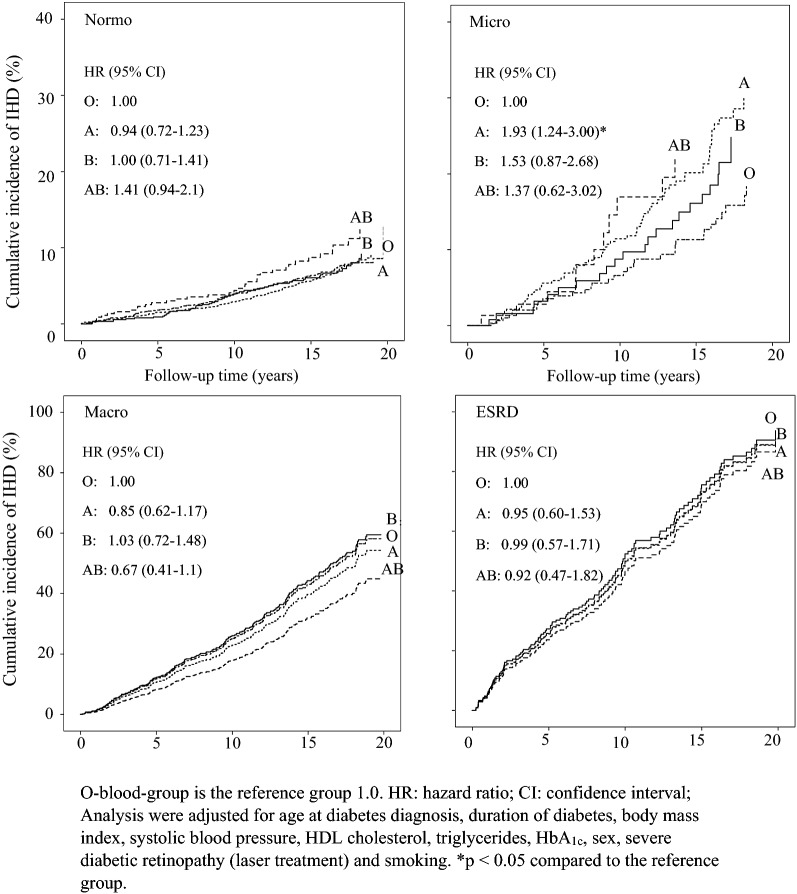
IHD risk of ABO blood groups stratified by DN stages

Among individuals with microalbuminuria, those with the A blood group showed the highest risk of IHD compared to those with O blood group (HR 1.93, CI 95% 1.24–3.00) (Fig. 2). A similar result was seen in the microalbuminuric group comparing the non-O blood group (A, B and AB) with the O blood-group (HR 1.81, CI 95% 1.15–2.84) (Additional file 1: Table S2). There was no difference in the risk of IHD between the ABO blood groups at any of the other DN stages (Fig. 2 and Additional file 1: Table S2).

**Fig. 3:**
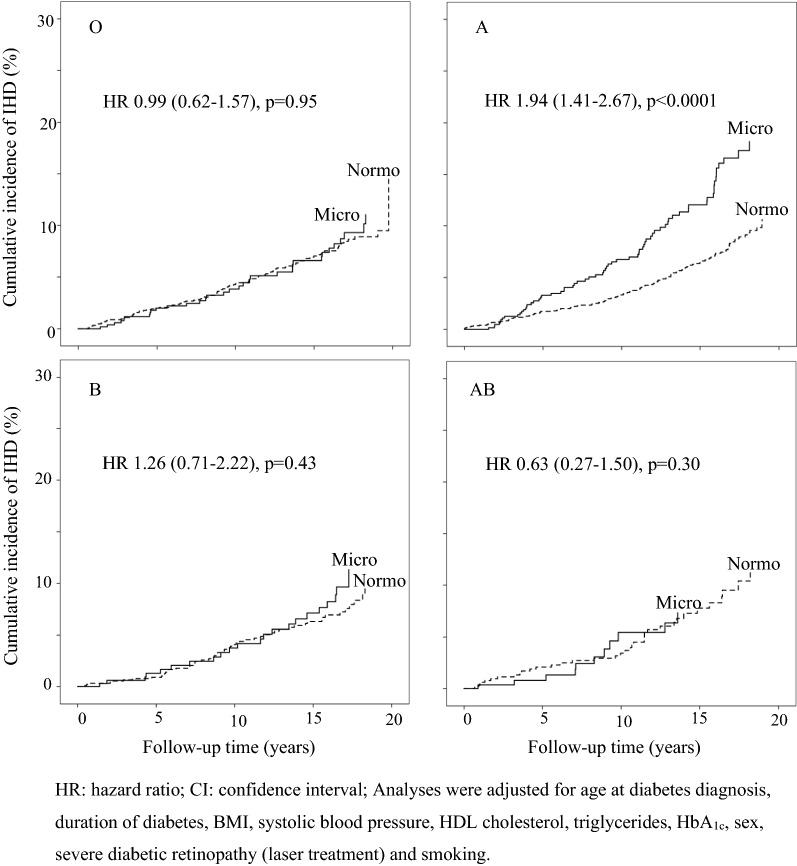
IHD risk between individuals with normo and microalbuminuria according to ABO blood groups

Comparing individuals with microalbuminuria to those with normoalbuminuria, only the A blood group showed a greater risk of IHD (HR 1.94, CI 95% 1.41–2.67, p < 000.1) (Fig. 3).

Among the individuals with the A blood group, the incidence of IHD events (29.3% vs 10.9%, p < 0.0001), levels of serum high sensitivity C-reactive protein (hs-CRP) (2.51 mg/l vs 1.71 mg/l, p < 0.001) and the number of antibiotic purchase per person per year (0.78 vs 0.47, p < 0.001) were greater in the individuals with microalbuminuria compared to those with normalbuminuria, although there was no difference in the prevalence of *FUT2* secretor phenotype (p = 0.82), nor the A1/A2 subtype distribution (p = 0.87) (Additional file 1: Table S3).

The risk of IS and PAD was no different among the ABO blood groups, regardless of DN status (Additional file 1: Tables S4, S5).

## Discussion

This is the first study to show that the A blood group is a risk factor for IHD in a large cohort of individuals with type 1 diabetes and microalbuminuria. The risk was 93% higher compared to the risk in those with the O blood group at the same DN stage. The risk of IHD was also 81% higher when the non-O blood group carriers were compared to the O blood group in those with microalbuminuria. Of note, the risk of the non-O blood group was driven by the high risk of the A blood group. Furthermore, individuals with microalbuminuria and the A blood group had a 94% higher risk of IHD compared to those with normoalbuminuria.

Although the O blood group has often been considered as the reference group with the lowest risk of ischemic CVD, the blood group with the highest risk has also varied depending on the studied population. Whether this is due to ethnical differences is not known. On one hand, a cross-sectional study with 299 individuals from Africa showed that the A blood group is the one with the highest risk of ischemic CVD, defined as coronary artery disease, myocardial infarction or IS [20]. On the other hand, a study on 64.686 blood donors from Canada showed that the AB blood group is the one carrying the highest risk of hospitalization or death due to thrombogenic events such as coronary, cerebrovascular or peripheral disease [21]. A study from Italy on a small cohort of 249 blood donors showed that the non-O blood group was the group with the highest risk of cardiovascular events [22], and similar results were obtained from a larger study from Sweden and Denmark with 1.5 million blood donors showing that individuals with the non-O blood group had higher incidence of both venous and arterial thromboembolic events than the O blood group [23]. Finally, a meta-analysis also described the A blood-group [2] and the non-O blood group [2, 3] as the highest risk groups for coronary artery disease, data that are in concordance with our results. However, the question arises, why does the A blood group in our and other populations confer an increased risk of IHD, and why is this risk particularly seen in those individuals with type 1 diabetes and microalbuminuria.

### ABO genotype and CVD

In order to answer this question we analyzed the ABO genotype distribution, especially the A1 and A2, among the different DN groups, since it has been described that the A1 subtype confers higher thrombogenic risk than the A2 subtype [23, 24], but we found no difference in the genotype distribution in our population. It has been suggested that the A blood group antigen can bind to endothelial cells and thereby contribute to cytoadherence, and the mechanism might involve antigen glycosylation that interacts with the p-selectin/ICAM-1 [25]. In this respect, the risk difference among the A blood group individuals could possibly be explained by lower levels of sICAM-1 in the A1 compared to the A2 carriers [9]. Unfortunately, we did not have access to any sICAM-1 measurements from our population.

### FUT2 secretor phenotype and CVD

We also evaluated the frequency of *FUT2* secretors and non-secretors, in order to explore the interactions between the ABO antigens and the endothelial adhesion molecules. It has been suggested that only people with a functional *FUT2* gene can secrete ABO antigens into the body fluids, a phenomenon characterizing the *FUT2* secretor phenotype [26]. However, we did not find any differences in the distribution of *FUT2* secretor phenotype among the different ABO blood groups nor between the A blood group individuals with normo- or microalbuminuria in our population.

### Infections and CVD

It is of note that there is a clear association between bacterial infections and the risk of CVD [27, 28], and it is also well-known that bacterial infections have both a direct and an indirect effect on the atherosclerotic process [27], an inflammatory condition that starts with lesions and dysfunction of the endothelium [28]. As previous data from our group showed that individuals with type 1 diabetes have a higher risk of bacterial infections [29], thus, we explored whether this increased risk of infections may be due to the ABO blood group and whether the ABO blood group could also have an impact on the risk of CVD in this population. Although there were no differences in the prevalence of infections among the various blood groups (Table 1), the individuals with the A blood group and microalbuminuria had a higher incidence of IHD events, higher hs-CRP levels and a higher number of antibiotic purchases per person per year compared to the individuals with normoalbuminuria with the same blood group. Thus, the inflammation/infection could be a possible mediator of this relationship between the A blood group, microalbuminuria and IHD. However, we do not know how inflammation after or during a bacterial infection, or how the microalbuminuric state might facilitate adhesion of the A-antigen to the endothelium.

### Lipids and CVD

Given the well-known associations between the lipids and CVD, we also investigated the distribution of total cholesterol, HDL-cholesterol and triglycerides among the ABO blood groups. However, there were no differences between the groups. In contrast, a Chinese study with 6476 individuals, showed that about 10% of the effect of the non-O blood group on the risk of coronary artery disease was mediated by its influence on LDL-cholesterol [30]. In an Indian study, the AB blood group was associated with high concentrations of HDL-cholesterol, while the O blood group was associated with low concentrations [11]. Besides these ethnical differences, there might be additional factors involved in type 1 diabetes such as proteomic alterations of the HDL-cholesterol [31] and a higher prevalence of coronary atherosclerosis compared to controls [32] that might increase the risk of CVD. Whether the ABO blood groups may have an impact on such alterations of the HDL-cholesterol molecules is not known.

### Other factors and CVD

There are also other factors that might interact with the ABO blood group antigens and thereby modulate the risk of ischemic CVD. For instance, it is well-known that the non-O blood group is associated with lower clearance of the vWF. Reduced clearance of vWF leads to a subsequent elevation of its plasma concentrations, which in turn enhances the chance of thrombogenic events [3, 10, 33]. Other explanations for the high risk of CVD in the non-O blood group could be the size of the platelets [34], the interaction between the red blood cell surface antigens (A, B, AB) with sICAM [3, 9, 25], p-selectin [25], E-selectin [35, 36] or plasma glycine [37, 38]. Unfortunately, none of these factors were measured in the present study.

### ABO blood groups and the risk of IS and PAD

In our analysis, the ABO blood groups were neither a relevant risk factor for IS nor PAD. Conversely, the MESA study showed that the A blood group was associated with a greater risk of PAD in African Americans, although it was not significant in Chinese, non-Hispanic white or Hispanic Americans [39]. Since our study was performed in a Caucasian Finnish population, race/ethnicity factors might be involved in the observed differences. Regarding the risk of IS, our results are similar to a Canadian study [21] in which any ABO blood group was a risk factor for hospitalization or death because of cerebrovascular disease. In the Canadian study, the ABO blood group was a risk factor for cerebrovascular disease only, when it was analyzed together with coronary artery disease. The smaller number of incident IS (n = 301) compared to the number of IHD (n = 893) could be a reason, why we did not detect any statistically significant difference in the IS risk.

Notably, in this study the ABO blood groups had no impact on CVD risk in individuals with advanced kidney disease, such as macroalbuminuria or ESRD. It is possible that advanced kidney disease in itself is such a strong risk factor for CVD that it might overrule any other thrombogenic risk factor, for instance the ABO blood groups.

### Limitations and strengths

A clear limitation is that we did not have information on vWF, sICAM-1, LFA-1, E-selectin, p-selectin, plasma glycine or platelet size that could have helped us further explore the relationship between the ABO blood groups and the thrombogenic risk. Another limitation is the small number of individuals in the AB blood group that limited our possibility to draw any conclusions regarding the CVD risk of this group.

Albeit these limitations, the strength of this study is its novelty to demonstrate that the A blood group is a risk factor for IHD in a large cohort of individuals with type 1 diabetes and microalbuminuria, a group that already carries a high CVD risk.

## Conclusion

The A blood group is an additional risk factor for IHD in individuals with type 1 diabetes and microalbuminuria, independently of the traditional CVD risk factors. In contrast, the ABO blood groups do not confer additional risk regarding IS or PAD in individuals with type 1 diabetes, regardless of the DN stage. Our results motivate further studies to elucidate the precise mechanism of the relationship between the A blood group, microalbuminuria and IHD. From a clinical perspective, our results raise the question, whether the ABO blood groups should be considered as an additional risk factor when CVD risk is assessed in individuals with type 1 diabetes and microalbuminuria.

## Supplementary information

**Additional file 1: Table S1.** Specific codes used for the cardiovascular outcomes from the relevant registries.**Table S2.** IHD risk of ABO blood groups stratified by nephropathy stages. **Table S3.** Comparison between individuals with normo and microalbuminuria with A-blood-group. **Table S4.** Ischemic stroke risk of ABO blood groups stratified by nephropathy stages. **Table S5.** Peripheral artery disease risk of ABO blood groups stratified by nephropathy stages. **Table S6.** List of physicians and nurses at each of the FinnDiane centers participating in patient recruitment and characterization.

## Data Availability

No data are available. The ethical statement and the informed consent do not allow for free data availability.

## Abbreviations

CVD: Cardiovascular disease
IHD: Ischemic heart disease
IS: Ischemic stroke
PAD: Peripheral artery disease
sICAM-1: Soluble InterCellular Adhesion Molecule 1
vWF: von Willebrand factor
DN: Diabetic nephropathy
UAER: Urinary albumin excretion rate
ESRD: End-stage renal disease
SD: Standard deviation
IQR: Interquartile ranges
HR: Hazard ratio
CI: Confidence interval
BMI: Body mass index
SBP: Systolic blood pressure
CRP: C-reactive peptide
LFA-1: Lymphocyte function-associated antigen-1
LDL: Low-density lipoprotein
HDL: High-density lipoprotein
