# Supplementary material for: Relationship between ABO blood groups and cardiovascular disease in type 1 diabetes according to diabetic nephropathy status
Source: Cardiovasc Diabetol. 2020 May 19;19:68. doi: 10.1186/s12933-020-01038-z (PMC7238526; doi:10.1186/s12933-020-01038-z)
Supplement: Supplementary file 1 — Additional file 1: Table S1. Specific codes used for the cardiovascular outcomes from the relevant registries.Table S2. IHD risk of ABO blood groups stratified by nephropathy stages. Table S3. Comparison between individuals with normo and microalbuminuria with A-blood-group. Table S4. Ischemic stroke risk of ABO blood groups stratified by nephropathy stages. Table S5. Peripheral artery disease risk of ABO blood groups stratified by nephropathy stages. Table S6. List of physicians and nurses at each of the FinnDiane centers participating in patient recruitment and characterization. [file 12933_2020_1038_MOESM1_ESM.docx]

Table S1. Specific codes used for the cardiovascular outcomes from the relevant registries.

| Ischemic heart disease | ICD-8/9 codes 410-414; ICD-10 codes I20–I25  Nordic Classification of Surgical Procedure codes TFN 40, 50 , FN1AT, FN1BT, FN1YT, FNF, FNG, FNA, FNB, FNC, FND, FNE, before 1996: 5311-5315 |
| --- | --- |
| Any stroke  Ischemic stroke | ICD-8/9 codes 430-434; ICD-10 codes I60-I64  ICD-8/9 codes 433, 434; ICD-10 code I63 |
| Amputations of lower extremity^a^  Revascularization of lower extremity^b^ | ICD-8 codes N895, N896, N897  ICD-10 codes S78,S88,S98, T053-T056  Nordic Classification of Surgical Procedure codes NFQ10,20, NGQ10,20, NHQ10,20,30,40,48 before 1996: 9571-75  Nordic Classification of Surgical Procedure codes PDF05, 20-22; PDH50-55, 99; PDN05, 20-22; PDW99; PEF23-25; PEH 56,57; PEN 23-25; PEW99; PGH41-47,49; PFH58-67; PDQ05, 20-22; PD1AT; PD1BT; PD1YT; PD3AT; PD3BT;PEQ23-25; PEQ99; PE1AT; PE1BT; PFQ26; PFQ99; PF1AT; PF1BT  before 1996: 9575, 5561-5568, 5579, 5581, 5583 |

Table S2. IHD risk of ABO blood groups stratified by nephropathy stages

|  | O | A | B | AB |
| --- | --- | --- | --- | --- |
| Normoalbuminuria, HR (95% CI)  n | 1.0 | 1.00 (0.77-1.31) | | |
|  | 1.0  (862) | 0.94 (0.72-1.23)  (1311) | 1.00 (0.71-1.41)  (505) | 1.41 (0.94-2.10)  (253) |
| Microalbuminuria, HR (95% CI)  n | 1.0 | 1.81 (1.15-2.84) * | | |
|  | 1.0  (178) | 1.93 (1.24-3.00) *  (251) | 1.53 (0.87-2.68)  (109) | 1.37 (0.62-3.02)  (44) |
| Macroalbuminuria, HR (95% CI)  n | 1.0 | 0.83 (0.62-1.11) | | |
|  | 1.0  (199) | 0.85 (0.62-1.17)  (272) | 1.03 (0.72-1.48)  (124) | 0.67 (0.41-1.10)  (55) |
| ESRD, HR (95% CI)  n | 1.0 | 0.95 (0.62-1.45) | | |
|  | 1.0  (94) | 0.95 (0.60-1.53)  (178) | 0.99 (0.57-1.71)  (58) | 0.92 (0.47-1.82)  (38) |

O-blood-group is the reference group 1.0. HR: hazard ratio; CI: confidence interval; Analysis were adjusted for age at diabetes diagnosis, duration of diabetes, body mass index, systolic blood pressure, HDL cholesterol, triglycerides, HbA_1c_, sex, severe diabetic retinopathy (laser treatment) and smoking. *p < 0.05 compared to the reference group.

Table S3. Comparison between individuals with normo and microalbuminuria with A-blood-group

|  | Normo  albuminuria  n=1311 | Micro  albuminuria  n=251 | p  value |
| --- | --- | --- | --- |
| IHD incident (n,%) | 139 (10.9) | 71 (29.3) | <0.0001 |
| FUT2 Secretor (%) | 82.2 | 82.8 | 0.82 |
| Hs-CRP (mg/l) | 1.71 (0.98-3.58) | 2.51 (1.26-5.33) | < 0.0001 |
| Antibiotic purchases per person year | 0.47 (0.23-0.95) | 0.78 (0.36-1.75) | < 0.0001 |
| A1A1 (%) | 10.0 | 10.8 | 0.87 |
| A1A2 (%) | 10.1 | 11.2 |  |
| A1O (%) | 52.4 | 48.6 |  |
| A2A2 (%) | 2.7 | 2.8 |  |
| A2O (%) | 24.8 | 26.5 |  |

Data are shown as percentage or median and interquartile range. P-value refers to χ2-test or Kruskal-Wallis test. IHD: ischemic heart disease; FUT2: fucosyltransferase-2; Hs-CRP:  high-sensitivity C-reactive protein.

Table S4. Ischemic stroke risk of ABO blood groups stratified by nephropathy stages.

|  | O | A | B | AB |
| --- | --- | --- | --- | --- |
| Normoalbuminuria, HR (95% CI) | 1.0 | 1.59 (0.95-2.66) | | |
|  | 1.0 | 1.60 (0.93-2.76) | 1.51 (0.77-2.95) | 1.74 (0.76-4.00) |
| Microalbuminuria, HR (95% CI) | 1.0 | 1.11 (0.61-2.01) | | |
|  | 1.0 | 1.02 (0.53-1.96) | 1.65 (0.79-3.45) | 0.49 (0.11-2.16) |
| Macroalbuminuria, HR (95% CI) | 1.0 | 1.12 (0.70-1.79) | | |
|  | 1.0 | 0.89 (0.52-1.52) | 1.64 (0.93-2.89) | 1.09 (0.50-2.35) |
| ESRD, HR (95% CI) | 1.0 | 0.94 (0.50-1.76) | | |
|  | 1.0 | 1.05 (0.54-2.04) | 1.00 (0.42-2.35) | 0.47 (0.13-1.69) |

O-blood-group is the reference group 1.0. HR: hazard ratio; CI: confidence interval; Analysis were adjusted for age at diabetes diagnosis, duration of diabetes, body mass index, systolic blood pressure, HDL cholesterol, triglycerides, HbA_1c_, sex, severe diabetic retinopathy (laser treatment) and smoking. p < 0.05 was considered significant for the comparison with the reference group.

Table S5. Peripheral artery disease risk of ABO blood groups stratified by nephropathy stages.

|  | O | A | B | AB |
| --- | --- | --- | --- | --- |
| Normoalbuminuria, HR (95% CI) | 1.0 | 1.00 (0.63-1.60) | | |
|  | 1.0 | 0.99 (0.60-1.65) | 0.84 (0.42-1.67) | 1.40 (0.63-3.12) |
| Microalbuminuria, HR (95% CI) | 1.0 | 0.86 (0.51-1.45) | | |
|  | 1.0 | 1.00 (0.57-1.75) | 0.70 (0.33-1.46) | 0.62 (0.21-1.84) |
| Macroalbuminuria, HR (95% CI) | 1.0 | 1.13 (0.77-1.67) | | |
|  | 1.0 | 1.10 (0.72-1.69) | 1.10 (0.66-1.84) | 1.30 (0.72-2.36) |
| ESRD, HR (95% CI) | 1.0 | 0.80 (0.49-1.31) | | |
|  | 1.0 | 0.67 (0.38-1.16) | 1.21 (0.65-2.25) | 0.73 (0.32-1.63) |

O-blood-group is the reference group 1.0. HR: hazard ratio; CI: confidence interval; Analysis were adjusted for age at diabetes diagnosis, duration of diabetes, body mass index, systolic blood pressure, HDL cholesterol, triglycerides, HbA_1c_, sex, severe diabetic retinopathy (laser treatment) and smoking. p < 0.05 was considered significant for the comparison with the reference group.

Table S6. List of physicians and nurses at each of the FinnDiane centers participating in patient recruitment and characterization
